# Renal hybrid oncocytic/chromophobe tumor associated with multiple schwannomas: Case report and literature review

**DOI:** 10.1097/MD.0000000000008939

**Published:** 2017-12-01

**Authors:** Guanghua Liu, Yatong Li, Zhuoran Li, Jingmin Zhou, Zhen Huo, Zhigang Ji

**Affiliations:** a Department of Urology; b Department of Pathology, Peking Union Medical College Hospital, Beijing, China.

**Keywords:** hybrid oncocytic/chromophobe tumor, kidney, oncocytoma, renal cell carcinoma, Schwannomas

## Abstract

**Rationale:**

Renal hybrid oncocytic/chromophobe tumors (HOCTs) are benign tumors containing a mixture of cells with features of chromophobe renal cell carcinoma (CHRCC) and renal oncocytoma (RO). Sporadic HOCT, which means HOCT occurs in patients without Birt–Hogg–Dubé syndrome (BHDS) or renal oncocytosis, is extremely rare. In this article, we would report a new case of a patient with both sporadic HOCT and multiple Schwannomas, which is even rarer than simplex sporadic HOCT.

**Patient concerns:**

A 48-year-old female was noted with multiple left-kidney masses and a history of multiple Schwannomas. She had no complaints of urological symptoms, abdominal pain, and osphyalgia. The vital sign was stable and blood biochemistry test showed normal renal function. Enhanced computed tomography (CT) found multiple lesions occupying parenchyma of the left kidney. The largest one was measured 3.5 × 3.1 × 3.2 cm. It showed apparently enhancement in arterial phase and low-density in venous phase.

**Diagnoses:**

The preoperative diagnosis was renal cell carcinomas.

**Interventions:**

The masses were removed by laparoscopic partial left nephrectomy.

**Outcomes:**

The diagnosis of HOCT was made by histopathology after surgery. No evidence of local recurrence or distant metastasis was noted on imaging after 2-month follow-up.

**Lessons:**

We searched PubMed for cases of sporadic HOCT and a total of 26 patients were evaluated. Our case was the first one involving sporadic HOCT and multiple Schwannomas. Although rare, sporadic HOCT does exist in patients presented with renal mass. Urological surgeons should be aware of the existence of HOCT when considering masses on kidney due to the different prognosis between HOCT and renal cell carcinoma. Further, a possible genetic relationship between HOCT and Schwannoma may contribute to a common pathogenesis in these 2 tumors.

## 1 Introduction

Renal hybrid oncocytic/chromophobe tumors (HOCTs) are relatively rare benign tumors containing a mixture of cells with features of chromophobe renal cell carcinoma (CHRCC) and renal oncocytoma (RO).^[1,2]^ It was reported that 15% to 18% of oncocytomas diagnosed by renal tumor biopsy were actually HOCT.^[3]^ The prognosis of HOCT is excellent. Most frequently, HOCTs occur in patients with Birt–Hogg–Dubé syndrome (BHDS)^[4]^ or renal oncocytosis.^[5]^ Schwannomas are benign tumors originated from peripheral nerve sheath. Only 1 case has been reported describing an association between HOCT and schwannomas in a patient with BHDS.^[6]^ Thus, it was extremely rare. However, here, we for the first time report an even rarer case in which both HOCT and schwannomas occur in a non-BHDS patient. We also review the previously reported sporadic HOCT cases and propose a possible relationship between HOCT and Schwannomas.

## 2 Case report

**Figure 1:**
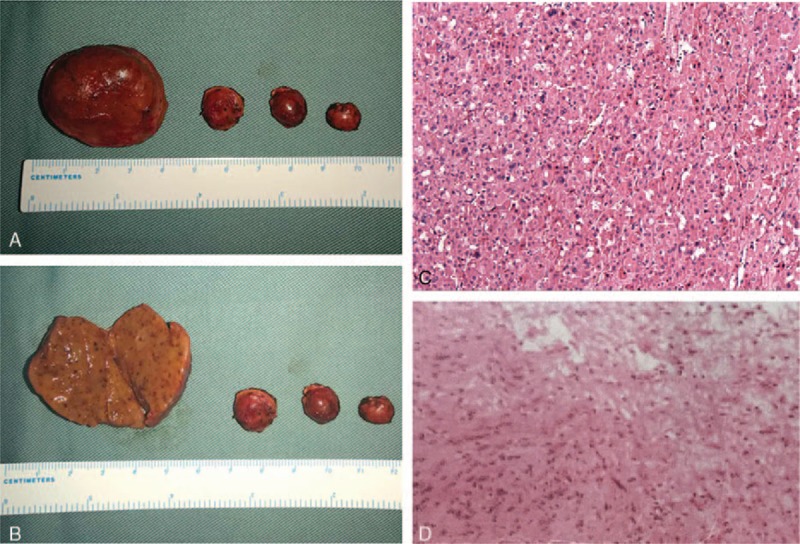
Histopathology of HOCT and schwannomas in our patient. (A)The surgical specimens of the renal masses. Four tumors were excised in total and the largest one was measured 4 × 3.5 × 3 cm. (B) The surgical specimens of 4 excised renal masses, with the largest one cut open. (C) HE staining of HOCT of left kidney in our patient (60×). (D) HE staining of Schwannoma in our patient (60×).

A 48-year-old female underwent B-ultrasound of kidney as a routine medical examination and she was found to have a well-circumscribed, solid, and hypoechoic mass located in the medial upper pole of the left kidney. She had no complaints of frequent micturition, urgent micturition, odynuria, abdominal pain, osphyalgia, and no sign of hematuria and proteinuria. She had a noteworthy past medical history that she underwent a mass excision located in the left lower quadrant of abdomen 30 years ago and a mass excision located in the left shoulder 6 years ago. Histopathological diagnosis confirmed both masses were schwannomas (Fig. 1D).

**Figure 2:**
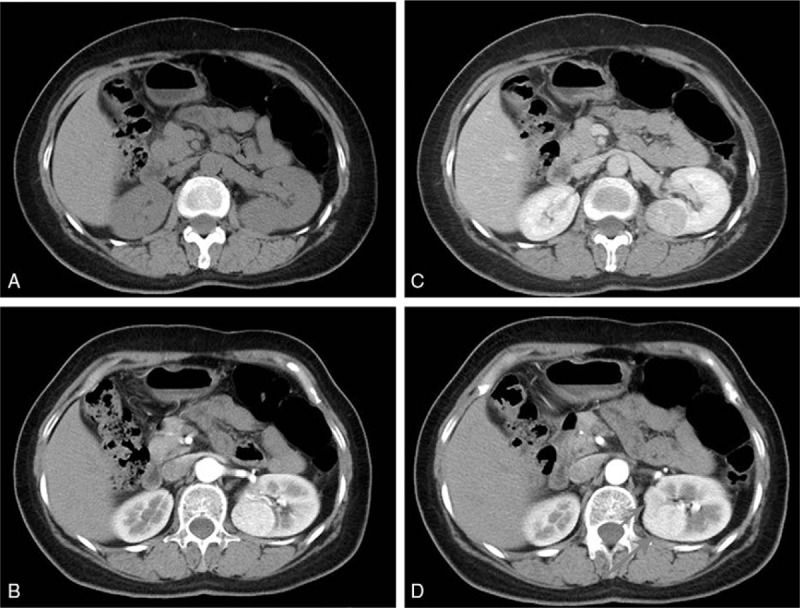
Preoperative CT of our patient. (A) Plain scan shows a large mass measured 3.5 × 3.1 × 3.2 cm in left kidney. (B) The mass has an apparently enhancement in arterial phase. (C) The mass shows a relatively low density compared with parenchyma in venous phase. (D) The multiple renal masses in arterial phase. The masses are pointed out by the red arrows.

Physical examination showed a stable vital sign and found no special physical sign in heart, lung, and abdomen. Blood biochemistry test showed normal renal function, including Cr (53 μmol/L), Urea (3.64 mmol/L), and uric acid (311 μmol/L). Urine routine test was also in normal limitation, including white blood cells (−), red blood cells (−), glucose (−), and cast (−). Enhanced computed tomography (CT) found multiple well-circumscribed quasi-round lesions occupying parenchyma of the left kidney. The largest one was measured 3.5 × 3.1 × 3.2 cm with an uneven density, located in the posterior lip at left renal hilar level, which showed marked enhancement in arterial phase and relatively low density in venous and delayed phase (Fig. 2). Left renal artery was pushed anteriorly by the mass with no marked constriction, while right renal artery and both renal veins showed no filling defect. Enhanced magnetic resonance imaging showed a quasi-round mass in the left kidney (3.6 cm in diameter) with an uneven enhancement. Renal blood flow diagram was performed and showed normal irrigation of both kidneys.

We considered renal cell carcinoma, renal hamartoma, and oncocytoma as our main differential diagnosis. RCC appears on CT as a solid mass with relatively uneven density (low, identical, or high) compared with renal parenchyma. Typically, it shows apparent enhancement in arterial phase and becomes a slightly low-density mass (compared with renal parenchyma) in delayed phase. Oncocytomas can be characterized on CT by a central-scar in a homogenous, well-circumscribed mass. However, it is usually difficult to distinguish oncocytomas with RCC based on imaging. Hamartomas can be reliably distinguished on CT as an enhancing mass that contains macroscopic fat and no calcification. As most renal hamartomas have a large proportion of fat, the lowest CT value should be negative, which is not consistent with our case. Renal Schwannomas were not included in our main concern because the prevalence was extremely low.

The preoperative diagnosis was renal cell carcinoma based on the prevalence, clinical features, radiographic findings, and the judgment of senior surgeons. The predetermined operation was radical nephrectomy considering the potential malignancy and multiple lesions. During surgery, a larger tumor located in the posterior lip of left renal hilar and 3 smaller tumors located in the inferior pole of left kidney were noted. All tumors were well-circumscribed and more likely to be benign lesions. Therefore, laparoscopic partial left nephrectomy was performed and all 4 noted lesions were completely excised (Fig. 1A, B). The largest resected renal tissue measured 4 × 3.5 × 3 cm. Hematoxylin and eosin staining was performed (Fig. 1C). The result of immunochemistry staining was positive for PAX-8, CA9, AE1/AE3, CD10, CK7 (partially), epithelial membrane antigen (EMA) (partially), and negative for P504, RCC, TFE3, and Vimentin. Consequently, a histopathological diagnosis of renal HOCTs was made.

The patient had an uneventful postoperative recovery. Cr (65 μmol/L) and Urea (3.93 mmol/L) demonstrated good renal function after surgery and she left hospital 5 days later. No evidence of renal impairment, local recurrence, or distant metastasis was noted after 2-month follow-up. The patient gave her informed consent for treatment and inclusion in this study, after having been provided with all the necessary information.

## 3 Discussion

HOCTs have been described in 3 conditions: patients with BHDS^[4]^; patients with renal oncocytosis^[5]^; and patients with no clinical features of these 2 diseases (sporadic).^[1,7]^ BHDS is an autosomal dominant disease characterized by skin fibrofolliculomas, together with an increased risk of pulmonary cysts, spontaneous pneumothorax, and renal cancer (especially CHRCC, RO, and HOCT).^[8]^ Renal oncocytosis is a condition in which bilateral kidneys are involved by diffused innumerable oncocytic nodules.^[5]^ Our patient had no sign of skin fibrofolliculomas and no history of spontaneous pneumothorax. Only unilateral kidney was involved by several nodules (4 nodules seen in the surgery). Therefore, we considered HOCT in our patient as sporadic.

To investigate sporadic HOCT, we searched PubMed using the keywords “hybrid oncocytic/chromophobe tumor,” “hybrid renal cell neoplasm,” and “sporadic.” The citation lists associated with all the studies retrieved were used to identify other potentially relevant publications. Finally, 26 nonrepeated cases of sporadic HOCT reported in 3 published articles were identified. The clinical features of these 26 cases are summarized in Table 1.^[9]^

**Table 1:**
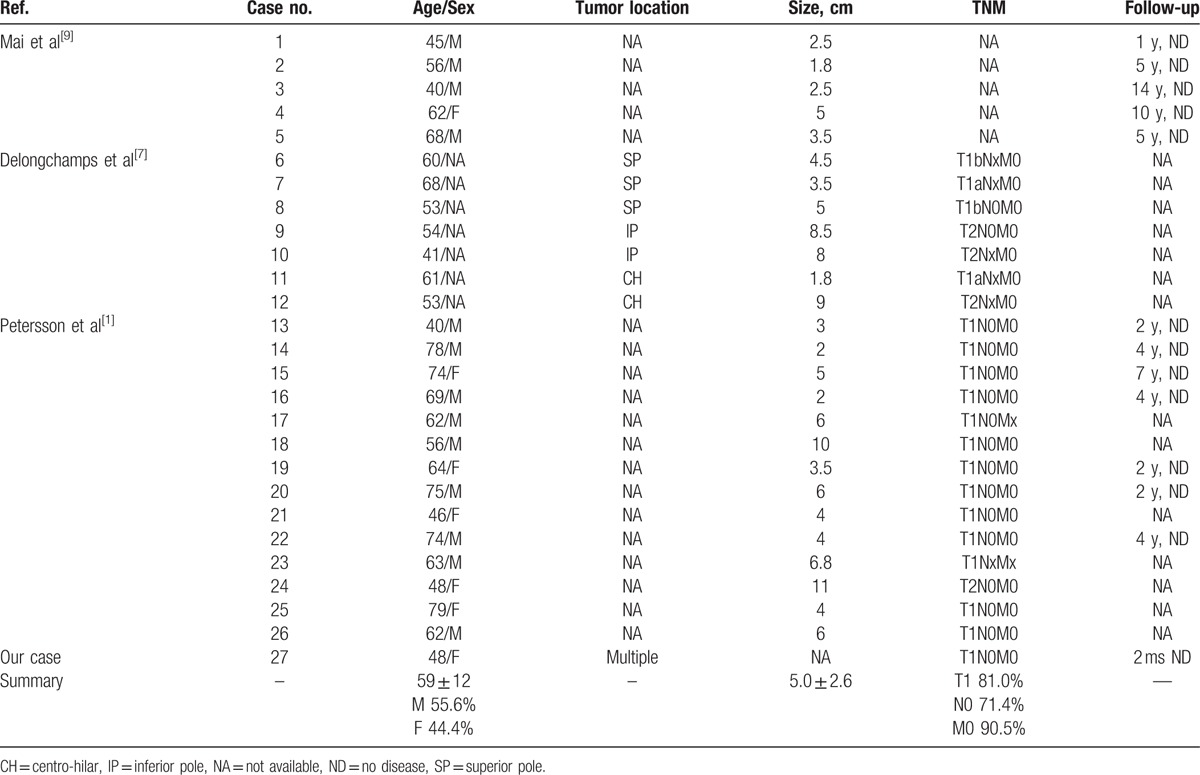
Summary of sporadic hybrid oncocytic/chromophobe tumors in all cases.

Among these patients, 15 (55.6%) were male and 12 (44.4%) were female. The mean age was 59 (range, 40–79) years. The mean size of tumor was 5.0 (range, 1.8–11) cm. No recurrence or metastasis was noted in the cases with available follow-up data. As Bhatnagar et al^[2]^ demonstrated, radiographic techniques such as CT may not be a reliable method to make a prospective diagnosis because HOCTs share similar features with other renal tumors, particularly RO and CHRCC. However, HOCTs do have some unique histopathological and molecular cytogenetic patterns. Petersson et al^[1]^ demonstrated that all sporadic HOCTs in their study were limited in the kidney with no infiltration of nearby tissue and distal metastasis, which showed a benign behavior, but that conclusion may be limited by the rare reports and relatively short follow-up. Tumors were composed of eosinophilic cells with perinuclear cytoplasmic clearing and there was not a distinct boundary between RO-like and CHRCC-like areas, which could also be noted in our hematoxylin and eosin staining figure. Immunochemical staining for sporadic HOCT was positive for AE1/AE3 and EMA and negative for Vimentin, which was consistent with our case. Delongchamps et al^[7]^ reported that no central scar could be noted in HOCT, which might be a character that distinguishes HOCT from RO. The prognosis of HOCT seemed to be excellent, as no recurrence and metastasis were noted according to Waldert et al^[3]^ and Delongchamps et al,^[7]^ with the mean follow-up of 50 and 20 months, respectively.

Schwannomas are well-circumscribed peripheral nerve sheath tumors containing a clonal population of Schwann cells. The biological behavior of Schwannomas is always benign. Only 1 case has been published describing the association between HOCT and multiple Schwannomas.^[6]^ However, to the best of our knowledge, our case was the first reported case where HOCT together with multiple Schwannomas occurred in the same patient without BHDS. Our case provided meaningful radiographic and histopathological materials of this disease, which might help surgeons realize the existence of the concurrence of sporadic HOCT and Schwannomas. Further, we also found a possible genetic relationship between renal tumors and Schwannomas. It has been reported that loss function of merlin (also known as schwannomin) accounts for the genesis of both sporadic and genetically acquired schwannomas.^[10]^ Recently, it was also noted that genetic changes in *NF2* (the gene that encodes merlin) might be common in patients with collecting duct carcinoma (CDC)-a subtype of renal cell carcinoma.^[11]^ On the basis of the evidence above, there might be a genetic association between sporadic HOCT and multiple Schwannomas, especially in the common genetic changes in NF2. However, molecular evidence is needed based on more cases reported in future.
